# Enhanced delivery of melatonin loaded nanostructured lipid carriers during *in vitro* fertilization: NLC formulation, optimization and IVF efficacy†

**DOI:** 10.1039/c9ra10867j

**Published:** 2020-03-04

**Authors:** Fatemeh Noori Siahdasht, Nafiseh Farhadian, Mohammad Karimi, Leili Hafizi

**Affiliations:** Chemical Engineering Department, Faculty of Engineering, Ferdowsi University of Mashhad Iran n.farhadian@um.ac.ir +9838806148 +9838805140; Department of Emergency Medicine, Faculty of Medicine, Ahvaz Jundishapur University of Medical Sciences Ahvaz Iran; Cardiovascular Research Center, School of Medicine, Mashhad University of Medical Sciences Mashhad Iran; Department of Obstetrics and Gynecology, Faculty of Medicine, Mashhad University of Medical Sciences Mashhad Iran

## Abstract

In this study, the potential of melatonin hormone loaded in nanostructured lipid carriers (Mel-NLCs) in the *in vitro* fertilization (IVF) environment is investigated by measuring the oocyte maturation, the two-pre-nucleus embryo development, the two-cell stage embryo development, and blastocyst production on the oocytes of mice. Mel-NLCs are prepared using the hot homogenization-ultrasonication method. A response surface method is utilized to determine the best independent variables to obtain nanoparticles with a small particle size and high hormone entrapment efficiency. The optimized nanoparticles have a particle size of 119 nm with a polydispersity index of 0.09 and hormone entrapment efficiency of 94%. Characterization results such as TEM and AFM analysis confirm the spherical and relatively uniform structure of the optimal sample. FTIR and XRD analyses indicate that the hormone is properly loaded within the amorphous nanostructure. Drug release from NLC under the *in vitro* environment exhibits a biphasic domain including burst release in the first 2 hours and the controlled release in 48 h 92% of the drug is released from nanoparticles in 48 hours, but the same amount of hormone is released from the marketed drug suspension during 2 hours. Results of IVF experiments reveal that the nanostructured form has a positive effect on all IVF parameters compared to the free form of the hormone. In addition, using the hormone nanostructured form can reduce the dosage of the melatonin free form with the same efficacy in the IVF environment. Finally, the nanostructured form of melatonin based on NLC nanostructure can be a good candidate for application in IVF media.

## Abstract 2

In this study, the potential of melatonin hormone loaded in nanostructured lipid carriers (Mel-NLCs) in the *in vitro* fertilization (IVF) environment is investigated by measuring the appropriate IVF parameters on the oocytes of mice.

## Graphical abstract

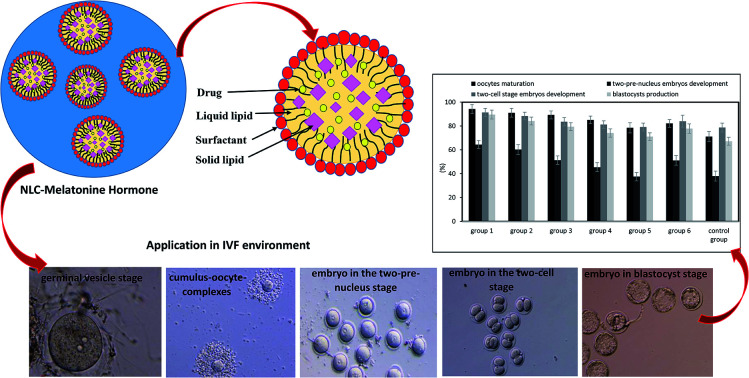

## 1. Introduction

Infertility is defined as the failure to conceive after one year of unprotected sexual intercourse.^1^ In light of advances in different fields of medical science, today infertility is no longer considered as an untreatable disease. *In vitro* fertilization (IVF) is a method of assisted reproductive technology (ART) that plays a vital role in this regard.^2^ Despite widespread progress in ART, the use of these methods has been limited due to a number of problems.^2–4^ The *in vitro* maturation (IVM) process may be hindered by the excessive generation of reactive oxygen species (ROS). These molecules include free radicals, peroxides and oxygen ions, which are highly reactive. ROS can damage the sperm membrane and oxidize DNA, lipids and proteins, resulting in mitochondrial dysfunction in gametes and embryos, low blastocyst production and inhibition of sperm fusion with the oocyte. Therefore, the addition of antioxidants such as l-carnitine, hyaluronan, resveratrol and melatonin can contribute to this process. Antioxidants can reduce ROS amounts and enhance blastocyst formation rates.^4–7^

Melatonin (*N*-acetyl-5-methoxytryptamine) is a neurohormone produced in the pineal gland with potent antioxidant properties, which can be used in the IVF as an auxiliary material.^8^ Therefore, melatonin can modulate ovarian function and reproduction in mammals.^9^ Melatonin and its metabolites can scavenge ROS and free radicals, preventing lipid peroxidation, protein and DNA damage. It is effective in enhancing the activity of antioxidant enzymes and reducing peroxidase enzymes.^10–13^ This molecule also promotes oocytes development, enhances the quality of *in vitro* development of embryos, preserves the optimal mitochondrial function and homeostasis, increases the cell numbers per blastocyst, raises the hatching blastocyst rates, improves mitochondria distribution, escalates cumulus cells expansion and enhances nuclear maturation rates.^5,11^ However, since melatonin is poorly soluble in aqueous solutions, it has a very short half-life of about 1.8–2.1 h,^14^ low bioavailability and distribution into cells. These specifications can restrict melatonin activity as an anti-apoptotic and anti-oxidant molecule.^5,6,15^

To date, a considerable number of drugs need to be administered frequently a day because of their short half-life in the body, which results in a significant fluctuation in the plasma drug concentration and drug toxic side effects. Therefore, the development of a sustained release oral dosage form instead of the conventional preparations for the side effect reduction and patient compliance improvement is desirable. Several approaches have been investigated to solve the aforementioned problems.^16^ One of the most applicable strategy to overcome these problems is to use nanocarriers. Nanocarriers provide a number of benefits such as the enhancement of bioavailability, the delivery of lipophilic drugs, the reduction of cytotoxicity and the improvement of antioxidant effects of drug. Thus, these systems allow molecular structures to be transferred directly into the oocyte during the fertilization process to produce transgenic offspring.^3,10^ Moreover, the particle size of nanocarriers has a significant role in the efficiency of drug delivery. For example, it is responsible for transmission from biological barriers, drug delivery to the subcellular sections and the controlled release of drugs.^5,6,17^

So far, melatonin has been prepared in the form of polymeric and lipid-core nanocapsules (LNC)^5,6^ for the IVF environment. Komninou *et al.*^5^ investigated the effectiveness of free melatonin, melatonin-loaded polymeric nanocapsules and melatonin-loaded lipid-core nanocapsules on *in vitro* cultured bovine embryos. They measured the protective effects of melatonin against oxidative stress and cell apoptosis during *in vitro* embryo culture in bovine species. They observed that lipid-core nanocapsules had smaller particle size than polymeric nanocapsules, with an average diameter of 171 nm and 237 nm, respectively. There was no difference between treatments on cleavage rates or in the proportion of oocytes that developed to 4-, 8-, 16-cell, morulae and blastocyst stages. However, the rates of hatched blastocysts in the Mel, and Mel-LNC-treated groups were higher than in the control groups.

Remião *et al.*^6^ treated oocytes with free melatonin, melatonin-loaded lipid-core nanocapsules and unloaded lipid-core nanocapsules during *in vitro* maturation. They evaluated cytotoxicity, meiotic maturation rate, development to the blastocyst stage, reactive oxygen species (ROS) and glutathione levels, mean cell number and apoptotic cell/blastocyst, and mRNA quantification. The mean hydrodynamic diameter of melatonin-loaded lipid-core nanocapsules was 181 nm. Free melatonin and melatonin-loaded lipid-core nanocapsules groups augmented *in vitro* embryo production. However, melatonin-loaded lipid-core nanocapsules group was more effective during *in vitro* oocyte maturation.

In both of the aforementioned studies, the particles size was relatively large. Therefore, the particle size reduction can increase melatonin effectiveness in the IVF environment. Smaller particles size make particles easier to cross biological barriers and deliver drugs better to the target site.^18^

One of the best nanoparticles for entrapment of hydrophobic drugs with low particle size is nanostructured lipid carrier (NLC). NLCs are a delivery system in which partial-crystallized lipid particles are dispersed in an aqueous phase containing emulsifier(s), as a potential delivery system may have some advantages in certain circumstances when compared with other colloidal carriers.^19^ NLCs have some unique properties such as small particle size, high drug loading capacity and drug expulsion reduction from the matrix during storage.^20–24^ However, NLC nanoparticles are safe due to the absence of organic solvents in their production. On the other hand, these nanocarriers are biocompatible. In addition, applying natural oils as lipid phase in NLC preparation could have synergetic effects on the IVF efficacy. Economically, it is very cost-effective and can be synthesized in a short time. The process of lipid nanoparticles formation is scalable and the final product has commercialization capability. These advantages make them ideal systems to encapsulate various therapeutic agents specially hydrophobic drugs in comparison to polymeric based nanomaterials.^25,26^

The aim of this study is to design and optimize a nanostructure form of melatonin based on lipid carriers. Nanostructured lipid carriers (NLC), which applied a natural oil like olive oil with excellent properties in pregnancy, was selected for this purpose. Changing material type and content can influence the particle size and drug entrapment efficiency. Hence, the experimental design based on response surface method was employed to predict the optimum nanocarriers. Then, drug release was investigated under *in vitro* environment comparable to the IVF media. After approving the controlled release nature of the nanostructure, its efficacy in the IVF environment was investigated.

## 2. Materials and methods

### 2.1. Materials

Melatonin was purchased from Ramopharmin Company (Iran). Glycerol monostearate (GMS) was obtained from Sigma Aldrich Company (USA). Polysorbate 80 (Tween 80) was supplied by Merck Company (Germany). Olive oil was purchased from the market. Pregnant mare serum gonadotropin (PMSG) was purchased from Organon Company (Netherlands). Tissue culture medium 199 (TCM), fetal calf serum (FCS) and bovine serum albumin (BSA) were supplied by Sigma Aldrich Company (USA). NMRI mice were provided by Razi Vaccine and Serum Institute (Iran).

### 2.2. Preparation of melatonin-loaded NLCs

Melatonin loaded in nanostructured lipid carrier (NLC) was prepared using the hot homogenization-ultrasonication method.^27^ In this method, solid and liquid lipids were mixed and heated at 75 °C. A homogeneous mixture was created by a probe sonicator. Melatonin was added to the hot lipid phase (428 mg) and stirred completely to fully dissolve the drug in lipids. Tween 80 was dissolved in 7 ml deionized water to prepare the aqueous phase and heated up to 75 °C. In the next step, the hot aqueous phase was added to the drug-lipid matrix and dispersed homogeneously using a probe sonicator for 2 min at 80 W. In this step, oil in water (O/W) nanoemulsion was generated. Finally, nanoemulsion was immediately cooled to form NLC. The NLC solution was frozen with liquid nitrogen and placed in a freeze/dryer device. The water contained in nanoparticles was removed after 48 h and dry powder was achieved.

In this study, olive oil was selected as the liquid lipid for its antioxidant, anti-inflammatory and antimicrobial properties, as well as its protective effect on heart and brain, pregnancy and breast feeding.^28^

### 2.3. Experimental design

There are several statistical models to design the nanoparticles formulation. In this study, response surface method (RSM) based on central composite design was employed to determine the appropriate range of parameters influencing the formulation of nanoparticles and optimization of this formulation.^29,30^ It allows evaluating the effect of several independent parameters on responses using a limited number of experiments.^31^

Here, independent variables were drug to lipid ratio (% w/w) (*A*), liquid lipid to total lipid ratio (% w/w) (*B*), and surfactant to lipid ratio (% w/w) (*C*). Responses consisted of mean particle size (*Y*_1_) and drug entrapment efficiency (*Y*_2_). Table 1 shows 5 levels of these variables. 20 tests were designed by the RSM method to determine the most important statistical parameters and to optimize the preparation of melatonin-loaded NLC. The goal of optimizing statistical parameters was to prepare a carrier with higher entrapment efficiency and a small particle size. Meanwhile, Design Expert 7.0.0 software was utilized to determine the response pattern. The suitable model for each variables was determined based on one-way analysis of variance (ANOVA) results.

Variables with respective coded levels of the central composite design for the preparation and optimization of melatonin-loaded NLC

**Table:** 

Independent variables	Level				
	−2	−1	0	+1	+2
*A*: drug to lipid ratio	0.49	1	1.75	2.5	3.01
*B*: liquid lipid to total lipid ratio	10	14	20	26	30
*C*: surfactant to lipid ratio	9.82	18	30	42	50.18

**Table:** 

Dependent variables	Constraints
*Y*_1_: mean particle size	Minimized
*Y*_2_: drug entrapment efficiency	Maximized

### 2.4. Characterization

The mean particle size and polydispersity index (PDI) of the optimized samples were measured using the particle size analyzer (PSA) (Vasco3, Cordouan, France). The zeta potential of samples was determined by zeta potential device (Zeta Compact, CAD, France).

The morphological characteristics of optimized NLC were investigated by TEM (Leo 912 AB, USA) at 150 kV. For this purpose, a 10-fold dilution of the sample was produced by distilled water. An specific amount of the diluted sample was put on the mesh copper grid coated with activated carbon, and then dried. Before observation, this sample was negatively stained with uranyl acetate (2%).^15^

In addition, atomic force microscopy (AFM) was recruited to investigate the surface morphology of the optimal NLC using AFM device (Full, Ara Pazhouhesh, Iran). To take images of the prepared NLCs, the solution was diluted 10 times and the quantitative amount of the diluted sample was put on the microscope slide. Then, this film was dried and imaging was performed.

Fourier transform infrared (FTIR) spectra of melatonin, GMS and optimized melatonin-loaded NLCs were recorded by FTIR spectrophotometer (Thermo Nicolet, USA). The recorded spectrums were in the range of 400 to 4000 cm^−1^.

The X-ray diffraction (XRD) study of optimal samples was conducted by an X-ray diffractometer (Bruker D8 Advance, Germany), using Cu Kα radiation wavelength of 1.54060 Å. The scanning was carried out at 2*θ* (from 4 to 75°) with step size of 0.04° and divergence slit size of 1°.

### 2.5. Drug entrapment efficiency

The free melatonin content in the solution containing NLCs was calculated to determine the entrapment efficiency (EE%). For this purpose, first the solution was centrifuged at 14 000 rpm for 20 min. Then, the upper solution was passed through the cellulose acetate filter (0.45 μm). Finally, the free drug content was determined in the filtered solution through the HPLC analysis. The EE of nanoparticles was calculated from eqn (1):1
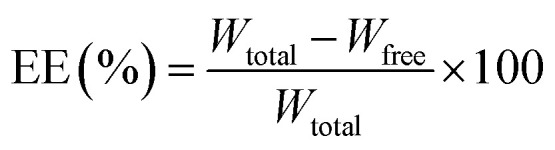
where *W*_total_ and *W*_free_ are the mass of initial melatonin and free melatonin in the aqueous phase, respectively.

The HPLC system consisted of pump, UV detector and C18 column (4.6 × 150 mm). The mobile phase consisted of methanol and water mixture with the mole ratio of 89 : 11. The flow rate and injection volume were 1 ml min^−1^ and 20 μl, respectively. The column effluent was monitored at 278 nm. A schematic of HPLC curve with the standard calibration curve within the concentration range of 7.81–500 mg l^−1^ is shown in ESI (see Fig. S1†). The retention time of melatonin is about 1 min.

### 2.6. Storage stability studies

To investigate the storage stability of the optimized NLCs, the freeze-dried samples were hold into vials at 4 °C for 0, 1 and 6 months. After the specified times, 5 mg of nanoparticle powder was dissolved in 10 ml of distilled water and the solution was sonicated for 2 min. Then, the stability of samples was measured by determining the particle size and entrapment efficiency percentage.^32,33^

### 2.7. *In vitro* drug release study

*In vitro* drug release study was carried out for free and nanostructured forms of drug using the dialysis bag technique in a simulated environment similar to the IVF environment. The dialysis bag with 12–14 kDa molecular weight cutoff was soaked in deionized water for 12 hours. Then, 5 ml of melatonin suspension and 5 ml of melatonin-loaded NLCs solution with the same drug content were placed into a dialysis bag. Dialysis bag was incubated in 50 ml of the phosphate buffer (pH = 7.4) at 37 °C under constant shaking (120 rpm). At certain time points, 1 ml of samples were withdrawn from the release medium and replaced with fresh buffer. The concentration of melatonin was determined by the HPLC analysis.^34,35^

### 2.8. *In Vivo* study in IVF media

The NMRI mice were housed in a controlled room under standard temperature, humidity, nutrition and light conditions (12 hours of light–12 hours of darkness) for 2 weeks to adapt with the environmental conditions. Then, mice with 6 to 8 weeks of age who were in the fertility period were chosen. These mice were subcutaneously injected with 5 units of PMSG to stimulate ovulation. The mice were killed 46–48 hours after PMSG injection by the relocation of cervical vertebrae. Then ovaries were removed and immediately transferred to the tissue culture medium 199 (TCM), which contained 5% fetal calf serum (FCS), and was heated in an incubator. The ovaries were punctured by an insulin needle to obtain the cumulus-oocyte-complexes (COCs) and germinal vesicle (GV).

To evaluate the effects of nanostructured melatonin on *in vitro* maturation of oocytes, the immature oocytes (COCs + GV) were placed in the TCM medium that contained different dosages of nanostructured melatonin (1 μM, 10 μM, 100 μM, 10 nM and 100 nM) and free melatonin (100 μM). Then, the oocyte maturation rate was evaluated by the reverse microscope after 18 to 24 hours.

The mice were killed by the relocation of cervical vertebrae and the sperms were taken from the tail of epididymis. The sperms were incubated with the T6 medium supplemented with 15% bovine serum albumin (BSA) for 1–1.5 hours to obtain the necessary talent. Then a sperm concentration of 1 × 10^6^ per ml was placed in 50 μl drops of T6 medium (including 15% BSA), with each drop receiving four to five mature oocytes in the *in vitro* medium. The oocytes were washed 4–6 hours after oocyte insemination with sperms in a clean T6 medium, and the two-pre-nucleus embryos were separated by the inverted microscopy and finally incubated. The two-pre-nucleus embryos development was investigated 18 hours after incubation in each group.

To evaluate the effect of nanostructured melatonin on the formation rates of blastocyst, the two-pre-nucleus embryos derived from *in vitro* fertilization treatments were divided into seven treatment groups. Then, embryo development was evaluated before the blastocyst stage.

The results were analyzed with Tukey test using SPSS 16 software. Evaluated groups were as follows:

Group 1: nanostructured drug with a concentration of 100 μM

Group 2: nanostructured drug with a concentration of 10 μM

Group 3: nanostructured drug with a concentration of 1 μM

Group 4: nanostructured drug with a concentration of 100 nM

Group 5: nanostructured drug with a concentration of 10 nM

Group 6: free drug with a concentration of 100 μM

Group 7: control group

All protocols used in present study were in accordance with National Institutes of Health guide for the care and use of laboratory animals (NIH Publications No. 8023, revised 1978) and was approved by the Mashhad University of Medical Sciences (Mashhad, Iran).

## 3. Results and discussion

### 3.1. Statistical design analysis

#### 3.1.1. ANOVA analysis

Table 2 shows independent variables including drug to lipid ratio (*A*), liquid lipid to total lipid ratio (*B*), and surfactant to lipid ratio (*C*) with measured responses. As can be seen, the mean particle size of the melatonin-loaded NLCs varies from 120.2 nm to 215.6 nm. The entrapment efficiency of the melatonin-loaded NLCs varies from 50.5% to 94%. For the purpose of clarity, the results of ANOVA analysis for both responses are reported in Table 3. Moreover, mathematical equations were produced for two responses to evaluate the effect of parameters. The negative value of a parameter in mathematical equations indicates the reduction of its response and *vice versa*.^36,37^

**Table:** Experimental design with independent variables and responses of the dependent variables for the formulations of melatonin-loaded NLC

Run	*A*: drug to lipid ratio (% w/w)	*B*: liquid lipid to total lipid ratio (% w/w)	*C*: surfactant to lipid ratio (% w/w)	*Y*_1_: mean particle size (nm)	*Y*_2_: entrapment efficiency (%)
1	1	26	18	169.3	94
2	2.5	14	18	215.6	80.1
3	3.01	20	30	190.5	82.9
4	1.75	20	50.18	149.1	78.1
5	1.75	20	30	150	83.8
6	1.75	10	30	202.7	85.2
7	2.5	26	18	181.5	86.7
8	1	14	42	135.5	86.3
9	1.75	20	9.82	179.7	87
10	1	14	18	186.9	56.4
11	1	26	42	120.2	87.6
12	1.75	30	30	141.9	90
13	2.5	26	42	161.8	50.5
14	2.5	14	42	178.2	83.5
15	1.75	20	30	144.1	89.8
16	1.75	20	30	154	83.9
17	1.75	20	30	160.6	84.7
18	1.75	20	30	139.4	85.1
19	0.49	20	30	135	84.8
20	1.75	20	30	148	82

ANOVA for response variables

**Table:** 

Mean particle size (*Y*_1_)						
Source	SS	df	MS	*F*-Value	*p*-Value	Significant/non-significant
Model	11 076.86	9	1230.76	14.00	0.0001	Significant
*A*	3497.11	1	3497.11	39.77	<0.0001	Significant
*B*	2523.79	1	2523.79	28.70	0.0003	Significant
*C*	3200.39	1	3200.39	36.39	0.0001	Significant
*AB*	38.72	1	38.72	0.44	0.5220	Non-significant
*AC*	235.44	1	235.44	2.68	0.1328	Non-significant
*BC*	50.00	1	50.00	0.57	0.4682	Non-significant
*A* ^2^	356.01	1	356.01	4.05	0.0719	Non-significant
*B* ^2^	1003.99	1	1003.99	11.42	0.0070	Significant
*C* ^2^	444.48	1	444.48	5.05	0.0483	Significant
Residual	879.40	10	87.94			
Lack of fit	602.40	5	120.48	2.17	0.2070	Non-significant

**Table:** 

Model summary statistics							
SD	Mean	C.V.%	Press	*R* ^2^	Adj *R*^2^	Pred *R*^2^	Adeq *R*^2^
9.38	162.20	5.78	5021.38	0.9264	0.8603	0.5800	13.544

**Table:** 

Entrapment efficiency (*Y*_2_)						
Source	SS	df	MS	*F*-Value	*P*-Value	Significant/non-significant
Model	1879.13	9	208.79	10.56	0.0005	Significant
*A*	52.18	1	52.18	2.64	0.1353	Non-significant
*B*	30.99	1	30.99	1.57	0.2390	Non-significant
*C*	43.12	1	43.12	2.18	0.1705	Non-significant
*AB*	533.01	1	533.01	26.96	0.0004	Significant
*AC*	396.21	1	396.21	20.04	0.0012	Significant
*BC*	720.10	1	720.10	36.43	0.0001	Significant
*A* ^2^	43.65	1	43.65	2.21	0.1681	Non-significant
*B* ^2^	2.48	1	2.48	0.13	0.7303	Non-significant
*C* ^2^	69.75	1	69.75	3.53	0.0898	Non-significant
Residual	197.68	10	19.77			
Lack of fit	162.97	5	32.59	4.70	0.0574	Non-significant

**Table:** 

Model summary statistics							
SD	Mean	C.V.%	Press	*R* ^2^	Adj *R*^2^	Pred *R*^2^	Adeq *R*^2^
4.45	82.12	5.41	1283.34	0.9048	0.8192	0.3821	13.602

The quadratic model was the best-fitted equation for the mean particle size response, as shown below:

2 Mean particle size (nm) = 153.38 + 16*A* – 13.59*B* − 15.31*C* + 7.85*B*^2^ + 5.06*C*^2^

where *A*, *B* and *C* represent the coded values of the drug to lipid ratio, liquid lipid to total lipid ratio and surfactant to lipid ratio, respectively. The results of ANOVA for the mean particle size response are depicted in Table 3. According to the table, this model was significant for response variables and insignificant for the lack of fit. The model accuracy can be evaluated by values of *R*^2^ and adjusted-*R*^2^. The values of *R*^2^ and adjusted-*R*^2^ were relatively the same and acceptable for this model, suggesting the reliability of the model in accurate prediction of results.

For the entrapment efficiency response, the quadratic model was the best-fitted, as shown below:

3 Entrapment efficiency (%) = 82.12 − 1.95*A* + 1.51*B* − 1.78*C* − 8.16*AB* − 7.04*AC* − 9.49*BC*

The results of ANOVA for the entrapment efficiency response are revealed in Table 3. According to the table, this model was significant for the response variable and insignificant for the lack of fit. Moreover, the data analysis demonstrated satisfactory values of *R*^2^ and adjusted-R^2^ in the model. The “Adeq Precision” factor represents the signal-to-noise ratio of the model, which is deemed acceptable for values higher than 4.^38^ The values of “Adeq Precision” for the entrapment efficiency and particle size responses are 13.602 and 13.544, respectively, which demonstrates the sufficiency of models for both responses.

#### 3.1.2. Interaction of variables

**Fig. 1:**
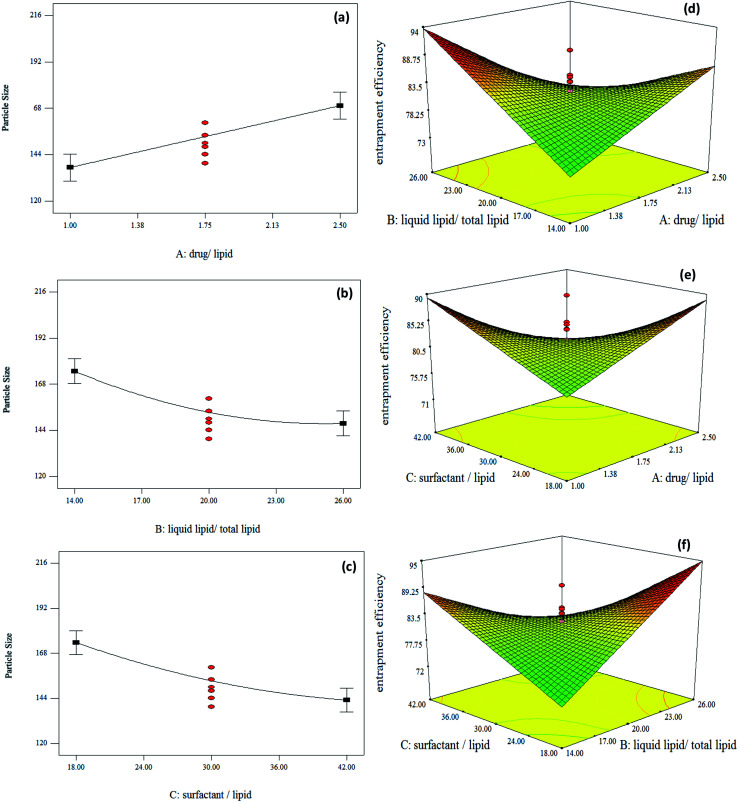
(a–c) One factor response plots for particle size and (d–f) three-dimensional response surface plots for drug entrapment efficiency.

The relationship between independent variables and responses are explained using the response surface analysis plots. As Table 3 shows, *A*, *B* and *C* parameters were significant for the particle size response, but their interactions (*AB*, *AC* and *BC*) were not significant. Thus, one factor plot was investigated for this response (Fig. 1).

As Fig. 1a shows, the particle size increases by enhancing the drug concentration. This is due to the melatonin incorporation in NLC matrix. By incorporating the drug into the NLC matrix, it is trapped inside the NLC structure and leads to its enlargement. A similar trend was observed by Emami *et al.*^39^ According to their results, changing the paclitaxel drug concentration from 5 to 10% increases the particle size. In addition, according to eqn (2), parameter *A* has the greatest effect on particle size.

The negative coefficient of *B* in eqn (2) indicates the reduction trend of particle size. The particle size of NLCs drops as the liquid lipid concentration increases (Fig. 1b), which is consistent with the results reported by Huang *et al.*^38^ It has been shown that the viscosity of melted GMS alone is higher than the viscosity of GMS and liquid lipid matrix. Therefore, this matrix of lipids can reduce viscosity and surface tension in the internal phase of NLC, which forms smaller nanoparticles.^40^

The surfactant to lipid ratio (*C*) was a parameter that influenced the size of NLCs. As Fig. 1c shows, the surfactant concentration enhancement reduced the particle size of NLCs. It is consistent with the findings of Zirak *et al.*^41^ who found that high surfactant concentration decreased the surface tension, and thus shrank the mean particle size.

According to Table 3, parameters *A*, *B* and *C* do not exert a significant effect on the entrapment efficiency response alone; however, the interaction of these parameters (*AB*, *AC* and *BC*) is significant. Hence, single parameters were remained in the model (eqn (3)). According to eqn (3), all interaction terms including *AB*, *AC* and *BC* had a negative effect on the entrapment efficiency.

As shown in Fig. 1d, at lower contents of liquid lipid (such as 14%), increasing drug to lipid ratio improves the entrapment efficiency. In the NLC structure, liquid lipid molecules are distributed between lipid matrix and nanoparticles with an imperfect structure that contain liquid and solid lipid mixture. This escalates drug entrapment in the structure. At higher contents of liquid lipid (*e.g.* 26%), increasing drug to lipid ratio diminishes entrapment efficiency due to reduced liquid lipid miscibility. Thus, the phase separation during cooling process leads to the rejection of drugs.

According to Fig. 1e, at higher contents of surfactants (*e.g.* 42%), entrapment efficiency falls by increasing drug to lipid ratio. At high surfactant concentrations, free molecules of surfactant start to form the micelle, which increases the hydrophobicity to hydrophilicity ratio of the solution. Since melatonin is a hydrophobic drug, it has good solubility in lipids and water phase at high concentrations of surfactant. Thus, the tendency of drug withdrawal from the lipid phase is boosted and its amount drops in nanoparticles. A similar behavior was observed by Elmowafy *et al.*^34^ They reported that higher solubility of the drug in the surfactant contributed to the drug removal from the oil phase and shrank its amount in the nanoparticles. However, at lower contents of surfactants (*e.g.* 18%), enhancing drug to lipid ratio increased entrapment efficiency. At lower concentrations of the surfactant, its molecules dissolve in solution, so they have a great ability in loading and maintaining drug molecules within the nanoparticle matrix.

As Fig. 1f depicts, at lower contents of liquid lipids (*e.g.* 14%), increasing the surfactant concentration boosts entrapment efficiency. However, when the content of liquid lipid is increased (*e.g.* 26%), the entrapment efficiency falls by elevated surfactant concentration.

#### 3.1.3. Optimization and validation

The optimized formulation was obtained using the numerical method prepared by Design Expert software. The input parameters were restrained in the range, whereas the desirability function of particle size and entrapment efficiency were based on the minimum and maximum level, respectively. The optimized melatonin-loaded NLCs were prepared by the formulation of a drug to lipid ratio of 1%, a liquid lipid to total lipid ratio of 26% and a surfactant concentration of 37.04%. The predicted values of particle size and entrapment efficiency were about 124 nm and 91% for optimal NLCs, respectively. The experimental values of the optimal NLC were comparable to the predicted values. Therefore, the credibility of models was confirmed by comparing experimental values (particle size of 119 nm and entrapment efficiency of 94%) with predicted values.

### 3.2. Characterization results

#### 3.2.1. Zeta potential and PDI value of Mel-NLCs

Given that zeta potential measures the surface charge density, it can predict the physical stability of the colloidal system. In other words, particle accumulation is less likely to happen when the zeta potential is high. When particles have the same pronounced electrical charge, the electrostatic repulsion occurs between them. It prevents particles from accumulation. Zeta potential and PDI values of the optimum formulation were estimated to be −18.56 mV and 0.09, respectively. The negative charge of zeta potential could be due to the presence of free fatty acids and the anionic nature of lipids.^42^ Electrostatic stability with zeta potential higher than −30 mV is desirable, as it engenders physical stability. However, this rule does not apply to systems that containing steric stabilizers, as these stabilizers reduce the zeta potential by shifting the shear plane of nanocarriers.^35,42^ Some stabilizers like Tween 80 can improve NLCs stability with their steric barrier properties. Therefore, the optimal sample used in the present study has a desirable zeta potential. Singh *et al.*^35^ observed a lower zeta potential in their optimal sample by applying Tween 80. Although the zeta potential was low, they stated that this amount was desirable.

#### 3.2.2. Particle size and morphology

**Fig. 2:**
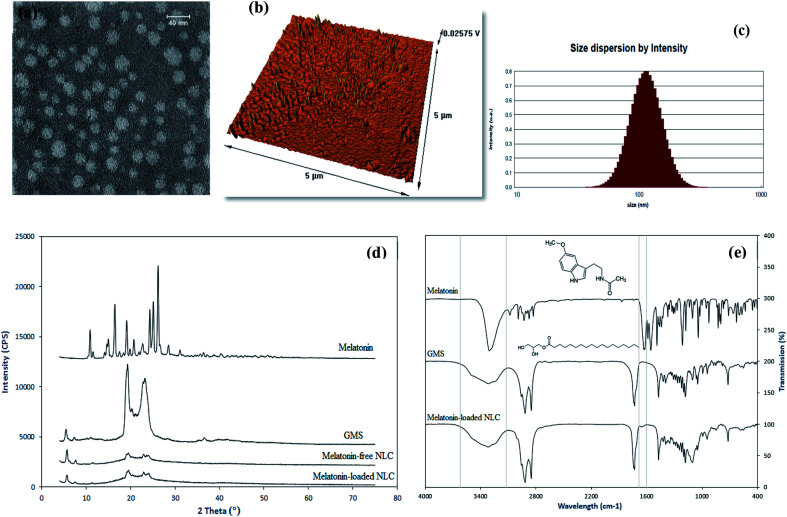
Characterization results of nanoparticles as: (a) TEM image, (b) AFM image, (c) PSA, (d) XRD patterns of melatonin, GMS, melatonin-loaded NLC and melatonin-free NLC, and (e) FTIR spectra of melatonin, GMS and melatonin-loaded NLC.

TEM (Fig. 2a) and AFM (Fig. 2b) analyses were carried out to investigate the morphology and size of the optimal melatonin-loaded NLCs. TEM image of the optimal sample exhibited spherical particles with a particle size of about 50 nm. The particles size measured by TEM was smaller than those obtained from Particle Size Analyzer (PSA) (Fig. 2c). These differences may be due to divergent measurement methods. The solvent layer attached to the particle improves the results of PSA.^35,38^ According to the AFM image, peaks have approximately the same height, which indicates the uniform distribution of particles in the optimal sample. These results are consistent with those reported by Huang and *et al.*^38^ In their study, NLC nanoparticles also had a spherical and uniform structure.

#### 3.2.3. X-ray diffraction study

To study the structure of melatonin-loaded NLCs, the XRD analysis was performed for pure melatonin, GMS, melatonin-loaded NLCs and melatonin-free NLCs. Fig. 2d shows the XRD patterns of samples, which were determined between angles 2*θ* = 4–75°. According to Fig. 2d, melatonin has sharp peaks at 10.86°, 16.41°, 19.08°, 24.30°, 25.06° and 26.15°, which exhibit the melatonin crystalline structure. GMS also has sharp peaks in the range of 18–24°, and therefore the GMS structure is also crystalline. However, as shown in Fig. 2d, the peaks of both melatonin-loaded NLCs and melatonin-free NLCs are wider with lower intensities compared to the GMS and pure melatonin. The results suggest that these nanocarriers have an amorphous structure. These differences can be due to the presence of other materials such as surfactants and liquid lipids in the NLCs structure.^42^ Also, all melatonin peaks do not manifest in the NLCs, which is in agreement with findings of Zhao *et al.*^43^ This may be due to the strong correlation between lipophilic drug and the lipid matrix, indicating that melatonin is well encapsulated into the NLCs and has an amorphous form.^42–44^

#### 3.2.4. FTIR analysis

FTIR analyses were performed for melatonin, GMS and melatonin-loaded NLC. The results are shown in Fig. 2e. The main peaks related to the GMS structure were observed at 3314 cm^−1^ (O–H), 2916 cm^−1^ and 2849 cm^−1^ (C–H), 1730 cm^−1^ (C=O), 1180 cm^−1^ and 1047 cm^−1^ (C–O).^45^ The principle peaks of melatonin were observed at 3303 cm^−1^ (N–H), 1629 cm^−1^ (C=O), 1555 cm^−1^ (C–O) and 1212 cm^−1^ (C–N).^46^ The NLC spectrum is comparable to that of GMS, which could be due to high lipid content compared to the amount of drug. This also demonstrates the successful placement of the drug into nanoparticle cavities, as a result of which some melatonin peaks are not visible in the spectrum of NLC and visible peaks are of lower intensity. The N–H stretching peak of melatonin is available in the NLC structure, but invisible due to the presence of a wide O–H peak of the GMS. However, amidic carbonyl group C=O stretching of melatonin is visible in the NLC structure. Therefore, the FTIR results confirm the presence of drug into the nanoparticle structure, which is consistent with XRD results. Topal *et al.*^46^ also observed similar results in their study. The melatonin complex spectrum in their research also had a few melatonin peaks, which proved the formation of an inclusion complex.

#### 3.2.5. Storage stability studies

The stability of freeze-dried sample was evaluated by measuring the mean particle size and entrapment efficiency after 0, 1 and 6 months. The mean particle size after 0, 1 and 6 months were 119.11 nm, 143.18 nm and 165.34 nm, respectively. Also, the entrapment efficiencies were calculated as 94.2, 93.1 and 90.2 at the preparation time and after 1 and 6 months, respectively. According to the results, the measured parameters of the samples were not considerably different during storage. The results suggested that the optimized sample retained its stability during storage.

### 3.3. *In vitro* drug release study

**Fig. 3:**
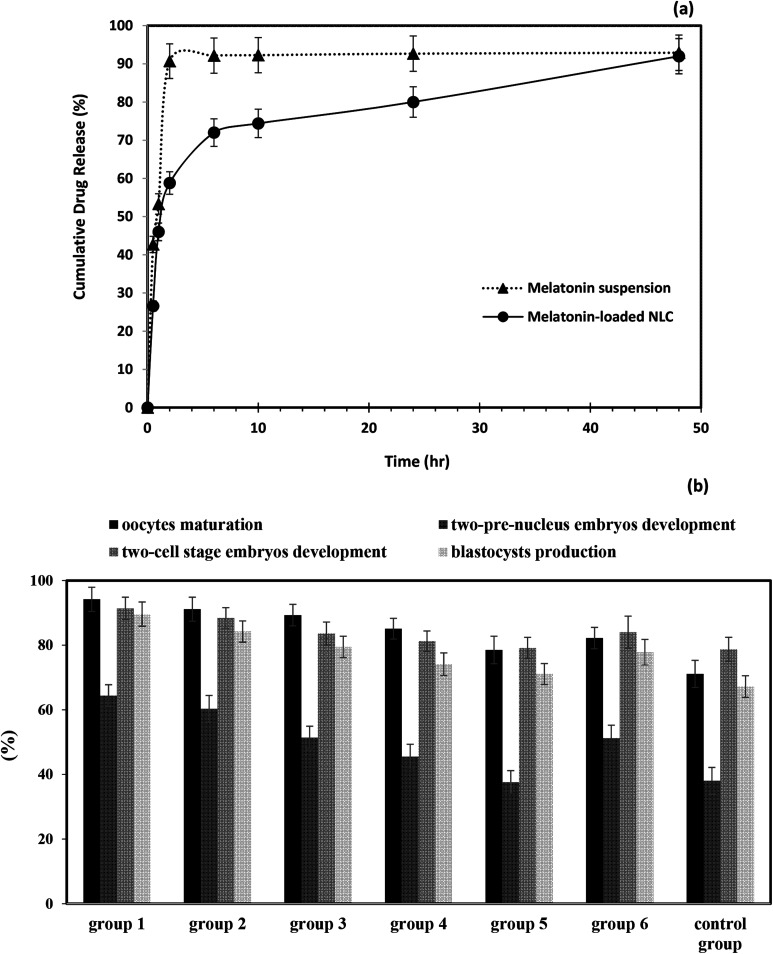
(a) *In vitro* drug release profile for melatonin suspension and melatonin-loaded NLCs and (b) effect of free melatonin and nanostructured form with different concentrations of drug various IVF parameters.

Fig. 3a shows drug release curves for melatonin suspension and melatonin-loaded NLCs. According to Fig. 3a, the drug release rate of melatonin-loaded NLCs was lower than that of melatonin suspension. The drug release from melatonin-loaded NLCs exhibits a biphasic domain including burst release in the early hours and controlled release in the later hours. In the early hours (2 h), the drug was released from the outer layer of NLCs, which led to the burst release.^47^ It was then followed by drug release from lipid matrix,^48^ which was associated with slow and controlled release for 48 h. However, the release rate of drug soared from suspension, so that 92% of drug was released in the first 2 h. Similarly, this drug release profile was observed for hydrophobic Dacarbazine drug in the NLC structure.^49^

### 3.4. IVF results

**Fig. 4:**
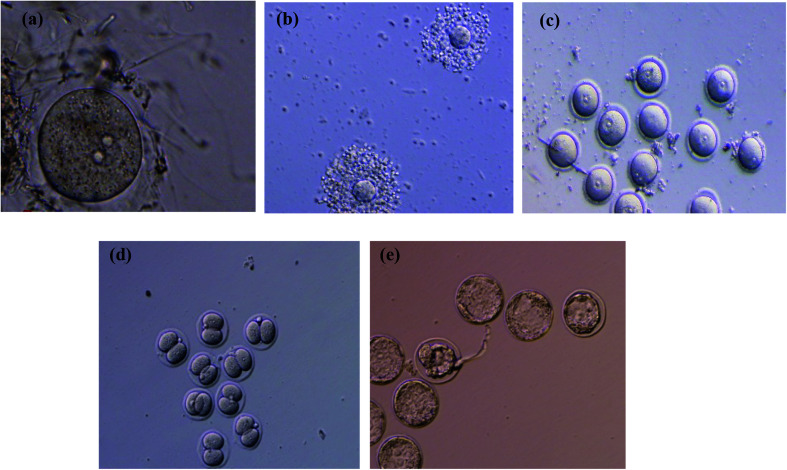
The oocyte and embryo images of the mice at different stages of maturation and development: (a) germinal vesicle stage (GV), (b) cumulus-oocyte-complexes (COCs), (c) embryo in the two-pre-nucleus stage, (d) embryo in the two-cell stage, and (e) embryo in blastocyst stage.

To evaluate the effectiveness of melatonin-loaded NLCs in the IVF environment, the effects of IVF treatments with free melatonin and nanostructured melatonin on oocyte maturation, two-pre-nucleus embryo development and two-cell stage embryo development as well as blastocyst production on oocytes of mice were investigated. The results of measured parameters for different studied groups are reported in Fig. 3b and Table S1 (see ESI file†). Fig. 4 shows the oocyte and embryo images of mice at different stages of maturation and development. Statistical analysis was performed using SPSS software to evaluate the effectiveness rate of different groups. Initially, the appropriate model was determined for the statistical analysis. One-way ANOVA analysis was selected since the data of all groups was normally distributed and their standard deviation was in a close range (see ESI file, Table S1†). According to the results of ANOVA, all parameters were significantly different between groups (*P* < 0.05). Given this significant difference, it is possible to compare mean values of the groups for each parameter. For this purpose, the homogeneity of variances test was examined initially. Since the variances were homogeneous in all cases (*P* > 0.05), the Tukey test was used to compare the groups (Table 4).

**Table:** The *p*-values obtained from comparing various groups

Comparison groups	*p*-Values of oocytes maturation	*p*-Values of the two-pre-nucleus embryos development	*p*-Values of the two-cell stage embryos development	*p*-Values of blastocyst production
**Free drug 100 μM with:**				
Nanostructured drug 100 μM	0.000	0.000	0.004	0.000
Nanostructured drug 10 μM	0.000	0.000	0.228	0.010
Nanostructured drug 1 μM	0.006	1.000	1.000	0.965
Nanostructured drug 100 nM	0.698	0.064	0.730	0.395
Nanostructured drug 10 nM	0.439	0.000	0.129	0.006
Control group	0.000	0.000	0.076	0.000

**Control group with:**				
Nanostructured drug 100 μM	0.000	0.000	0.000	0.000
Nanostructured drug 10 μM	0.000	0.000	0.000	0.000
Nanostructured drug 1 μM	0.000	0.000	0.126	0.000
Nanostructured drug 100 nM	0.000	0.005	0.811	0.004
Nanostructured drug 10 nM	0.004	1.000	1.000	0.298

**Nanostructured drug 100 μM with:**				
Nanostructured drug 10 μM	0.644	0.332	0.669	0.048
Nanostructured drug 1 μM	0.131	0.000	0.002	0.000
Nanostructured drug 100 nM	0.000	0.000	0.000	0.000
Nanostructured drug 10 nM	0.000	0.000	0.000	0.000

**Nanostructured drug 10 μM with:**				
Nanostructured drug 1 μM	0.953	0.000	0.146	0.111
Nanostructured drug 100 nM	0.030	0.000	0.005	0.000
Nanostructured drug 10 nM	0.000	0.000	0.000	0.000

**Nanostructured drug 1 μM with:**				
Nanostructured drug 100 nM	0.275	0.050	0.848	0.053
Nanostructured drug 10 nM	0.000	0.000	0.203	0.000

**Nanostructured drug 100 nM with:**				
Nanostructured drug 10 nM	0.014	0.002	0.911	0.587

The results of statistical analysis for various evaluated parameters can be discussed as follows:

#### 3.4.1. Effect of free and nanostructured melatonin on oocyte maturation

As Fig. 3b shows, when oocytes are incubated with the free and nanostructured melatonin, the oocyte maturation is significantly raised in all groups compared to the control group (*p* < 0.05). Also, the elevated concentration of nanostructured melatonin has a positive effect on the oocyte maturation, so that the first group has the highest oocyte maturation rate among all groups. Therefore, melatonin protects oocytes against reactive oxygen species (ROS) during oocyte maturation and increases maturation, which is in agreement with the results of Taketani *et al.*^11^

#### 3.4.2. Effect of free and nanostructured melatonin on two-pre-nucleus embryos development

The two-pre-nucleus embryos development is an criterion for successful *in vitro* fertilization of oocytes. Treatments with free and nanostructured melatonin at concentrations of 100 μM, 10 μM, 1 μM and 100 nM, dramatically increased the two-pre-nucleus embryos development of oocytes in comparison with the control group (*p* < 0.05). However, treatment with the nanostructured melatonin at a concentration of 10 nM did not show any significant difference with the control group (*p* > 0.05). Moreover, Group 1 had the highest rate of two-pre-nucleus embryos development compared to other groups (Fig. 3b).

#### 3.4.3. Effect of free and nanostructured melatonin on the two-cell stage embryos development

According to the results, the two-cell stage embryos development is significantly boosted as a result of treatments with nanostructured melatonin at concentrations of 100 μM and 10 μM, in comparison to the control group (*p* < 0.05). However, free and nanostructured melatonin at concentrations of 1 μM, 100 nM and 10 nM, were not significantly different from the control group (*p* > 0.05).

#### 3.4.4. Effect of free and nanostructured melatonin on blastocyst production

According to the results, the blastocyst production is dramatically increased through incubation of embryos with free and nanostructured melatonin at concentrations of 100 μM, 10 μM, 1 μM and 100 nM in comparison with the control group. However, nanostructured melatonin at concentration of 10 nM did not have any significant effect on blastocyst production (*p* > 0.05).

#### 3.4.5. Effect of melatonin concentration

According to the results, all measured parameters in the IVF environment were positively correlated with the concentration of nanostructured melatonin hormone. However, the first group (100 μM) had the highest impact among all groups and Group 5 (10 nM) had the lowest effect on various parameters, with a performance resembling that of the control group. Furthermore, the nanostructured melatonin at a concentration of 1 μM displayed a performance similar to that of free melatonin at a concentration of 100 μM. Therefore, a nanostructured form with a lower concentration of drug could be replaced in a free drug form with a higher concentration.

## 4. Discussion

In general, comparing the performance of free and nanostructured forms of melatonin suggests that Mel-NLC is highly effective in the IVF environment. This may due to higher hydrophilicity, smaller particle size and controlled release of hormone in the NLC form. Given the hydrophobicity property of melatonin, preparing the nanostructured form for this hormone facilitates the hormone release in the *in vitro* fertilization environment. In the other words, chaining the conventional form of melatonin to NLC structure could simultaneously improve its solubility and control its release. Therefore, release drug pass through biological membranes at longer time and produces higher uptake. In addition, the small size of nanocarriers and the controlled release of melatonin over time could boost its effectiveness by transference into the subcellular sections of the oocyte and embryo, where Mel-NLC remained until the final stages. Hence, it remain effectiveness until the last stages of *in vitro* fertilization. The main purpose of the controlled release systems is to maintain drug concentration in target tissues at a desired value as long as possible. They are able to exert a control on the drug release rate and duration. For this purpose, generally, controlled release system initially release part of the dose contained in order to attain rapidly the effective therapeutic concentration of the drug. Then, drug release kinetics follows a well defined behavior in order to supply the maintenance dose enabling the attainment of the desired drug concentration.^50^ So, it prolongs the duration of drug action.

Our observations in this study are in agreement with those reported by Remião *et al.*^6^ Remião *et al.* observed that Mel and lipid-core nanocapsules groups generated positive effects relative to the control group, leading to a higher blastocyst production rate. By comparing the rate of blastocyst production in our study and Remião *et al.* study at the same concentration of drug (1 μM), it can be observed that we could achieve the higher rate of blastocyst production (enhanced rate of 12% in our study and 9% in Remião *et al.* study). Maybe, the nature of nanostructured lipid carrier, sustained drug release behavior and lower particle size of NLCs could improve the blastocyst production comparing with lipid core nanocapsules.

Finally, it should be noted that although olive oil has many suitable properties, but it cannot change the results of IVF alone. Comparing the nanostructure group with the lowest melatonin dose (1 nM) and control group shows that there is not any remarkable difference between IVF parameters. Only, oocytes maturation has changed significantly. Maybe, applying olive oil could have some synergetic effects besides melatonin drug, but the melatonin concentration is more effective parameter than natural olive oil application.

## 5. Conclusions

This study represents the use of nanoencapsulated melatonin in the form of nanostructured lipid carriers during *in vitro* oocyte maturation. A smallest particle size and the highest drug entrapment efficiency were optimized by applying design of experiment approach. We demonstrated that sustained release of melatonin by encapsulating it into nanostructured lipid carrier could effectively improve the efficiency of bovine oocyte *in vitro* maturation, two-cell stage embryo development, and blastocyst production. We also found that treatment with Mel-NLC was superior to using conventional form of melatonin with regard to embryo quality, probably because this configuration facilitates the release of melatonin throughout the entirety of *in vitro* embryo production. Moreover, statistical analysis suggested that applying nanostructured hormones with lower concentration could be replaced with a free drug of higher concentration and similar effectiveness. In the future *in vivo* studies are planned.

## Conflicts of interest

There are no conflicts of interest to declare.

## Supplementary Material

RA-010-C9RA10867J-s001
