# Supplementary material for: Enhanced delivery of melatonin loaded nanostructured lipid carriers during in vitro fertilization: NLC formulation, optimization and IVF efficacy
Source: RSC Adv. 2020 Mar 4;10(16):9462–75. doi: 10.1039/c9ra10867j (PMC9050139; doi:10.1039/c9ra10867j)
Supplement: RA-010-C9RA10867J-s001 [file RA-010-C9RA10867J-s001.pdf]

Electronic Supplementary Material (ESI) for RSC Advances.
This journal is © The Royal Society of Chemistry 2020

Table S1. ANOVA results for evaluated parameters in the IVF environment

Parameters / Factors	Oocytes maturation		Two-pre-nucleus embryos development		Two-cell stage embryos development		Blastocyst production	
	Between Groups	Within Groups	Between Groups	Within Groups	Between Groups	Within Groups	Between Groups	Within Groups
Sum of Squares	3057.57	669.01	5076.11	715.56	1066.95	661.95	2835.54	595.56
df	6	49	6	49	6	49	6	49
Mean Square	509.60	13.65	846.02	14.60	177.82	13.51	472.59	12.15
F	37.32	-	57.93	-	13.16	-	38.88	-
Sig.	0.000	-	0.000	-	0.000	-	0.000	-
groups	Mean	Std. Deviation	Mean	Std. Deviation	Mean	Std. Deviation	Mean	Std. Deviation
(1) nanostructured drug 100 μM	94.21±1.31	3.72	64.43±1.18	3.35	91.39±1.23	3.46	89.61±1.32	3.75
(2) nanostructured drug 10 μM	91.14±1.31	3.70	60.29±1.47	4.16	88.41±1.13	3.21	84.22±1.16	3.28
(3) nanostructured drug 1 μM	89.30±1.18	3.33	51.38±1.26	3.55	83.62±1.25	3.55	79.45±1.17	3.31
(4) nanostructured drug 100 nM	85.09±1.31	3.20	45.51±1.35	3.82	81.23±1.12	3.16	74.13±1.23	3.47
(5) nanostructured drug 10 nM	78.52±1.50	4.26	37.60±1.26	3.57	79.13±1.17	3.30	71.08±1.14	3.24
(6) Free drug 100 μM	82.18±1.17	3.32	51.19±1.44	4.06	84.03±1.76	4.98	77.82±1.40	3.96
(7) Control group	71.11±1.48	4.20	38.02±1.47	4.15	78.70±1.32	3.74	67.19±1.18	3.34

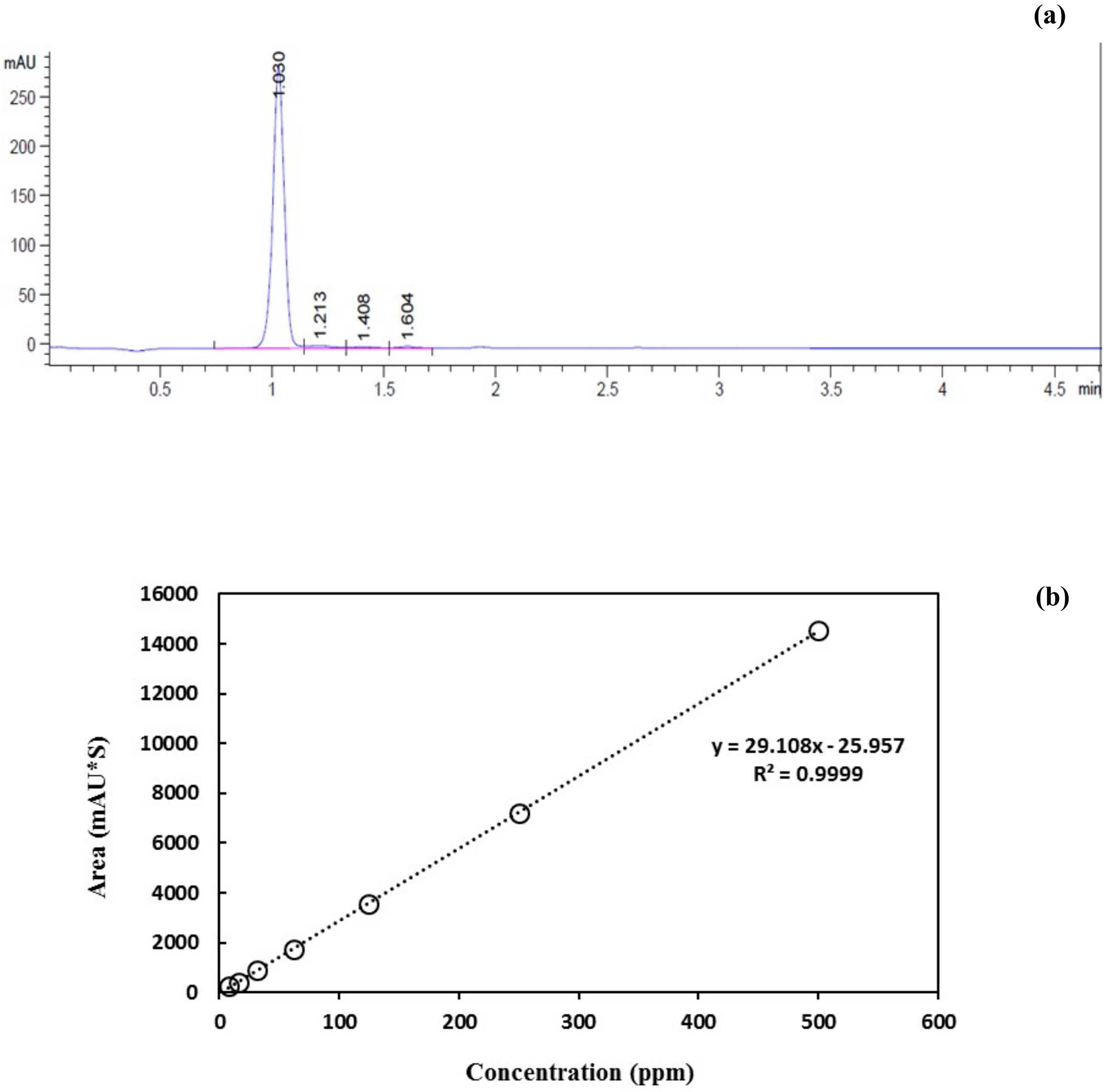

Figure S1. (a) The HPLC curve of melatonin hormone at the concentration of 7.81 ppm and (b) the standard calibration curve for melatonin
